# MBDBMetrics: an online metrics tool to measure the impact of biological data resources

**DOI:** 10.1093/bioadv/vbad180

**Published:** 2023-12-18

**Authors:** Giuseppe Insana, Alex Ignatchenko, Maria Martin, Alex Bateman, UniProt Consortium, Alex Bateman, Maria-Jesus Martin, Sandra Orchard, Michele Magrane, Shadab Ahmad, Emily H Bowler-Barnett, Hema Bye-A-Jee, Paul Denny, Tunca Dogan, ThankGod Ebenezer, Jun Fan, Leonardo Jose da Costa Gonzales, Abdulrahman Hussein, Alexandr Ignatchenko, Giuseppe Insana, Rizwan Ishtiaq, Vishal Joshi, Dushyanth Jyothi, Swaathi Kandasaamy, Antonia Lock, Aurelien Luciani, Jie Luo, Yvonne Lussi, Pedro Raposo, Daniel L Rice, Rabie Saidi, Rafael Santos, Elena Speretta, James Stephenson, Prabhat Totoo, Nidhi Tyagi, Preethi Vasudev, Kate Warner, Rossana Zaru, Supun Wijerathne, Khawaja Talal Ibrahim, Minjoon Kim, Juan Marin, Alan J Bridge, Lucila Aimo, Ghislaine Argoud-Puy, Andrea H Auchincloss, Kristian B Axelsen, Parit Bansal, Delphine Baratin, Teresa M Batista Neto, Jerven T Bolleman, Emmanuel Boutet, Lionel Breuza, Blanca Cabrera Gil, Cristina Casals-Casas, Elisabeth Coudert, Beatrice Cuche, Edouard de Castro, Anne Estreicher, Maria L Famiglietti, Marc Feuermann, Elisabeth Gasteiger, Sebastien Gehant, Arnaud Gos, Nadine Gruaz, Chantal Hulo, Nevila Hyka-Nouspikel, Florence Jungo, Arnaud Kerhornou, Philippe Le Mercier, Damien Lieber-herr, Patrick Masson, Anne Morgat, Ivo Pedruzzi, Sandrine Pilbout, Lucille Pourcel, Sylvain Poux, Monica Pozzato, Manuela Pruess, Nicole Redaschi, Catherine Rivoire, Christian J A Sigrist, Shya-mala Sundaram, Anastasia Sveshnikova, Cathy H Wu, Cecilia N Arighi, Chuming Chen, Yongxing Chen, Hongzhan Huang, Kati Laiho, Minna Leh-vaslaiho, Peter McGarvey, Darren A Natale, Karen Ross, C R Vinayaka, Yuqi Wang, Jian Zhang

**Affiliations:** European Molecular Biology Laboratory, European Bioinformatics Institute (EMBL-EBI), Wellcome Genome Campus, Hinxton CB10 1SD, United Kingdom; European Molecular Biology Laboratory, European Bioinformatics Institute (EMBL-EBI), Wellcome Genome Campus, Hinxton CB10 1SD, United Kingdom; European Molecular Biology Laboratory, European Bioinformatics Institute (EMBL-EBI), Wellcome Genome Campus, Hinxton CB10 1SD, United Kingdom; European Molecular Biology Laboratory, European Bioinformatics Institute (EMBL-EBI), Wellcome Genome Campus, Hinxton CB10 1SD, United Kingdom

## Abstract

**Motivation:**

There now exist thousands of molecular biology databases covering every aspect of biological data. This database infrastructure takes significant effort and funding to develop and maintain. The creators of these databases need to make strong justifications to funders to prove their impact or importance. There are many publication metrics and tools available such as Google Scholar to measure citation impact or AltMetrics covering multiple measures including social media coverage.

**Results:**

In this article, we describe a series of novel impact metrics that have been applied initially to the UniProt database, and now made available via a Google Colab to enable any molecular biology resource to gain several additional metrics. These metrics, powered by freely available APIs from Europe PubMedCentral and SureCHEMBL cover mentions of the resource in full text articles, including which section of the paper the mention occurs in, grant acknowledgements and mentions in patent applications. This tool, that we call MBDBMetrics, is a useful adjunct to existing tools.

**Availability and implementation:**

The MBDBMetrics tool is available at the following locations: https://colab.research.google.com/drive/1aEmSQR9DGQIZmHAIuQV9mLv7Mw9Ppkin and https://github.com/g-insana/MBDBMetrics.

## 1 Introduction

Databases of molecular biology data have become central to modern biology and many thousands of resources now exist (Ma *et al.* 2022). These range from extremely specialized databases that focus on perhaps a single aspect of biology in a single organism, or repositories of data from a single laboratory, up to huge international collections of data on nucleic acids, genetic variation or macromolecular structure. Continued funding or lack of it for these databases has been a constant theme over the last three decades and in more recent years there have been moves to coordinate international funding in this arena, from ELIXIR (Harrow *et al.* 2021) and the Global Biodata Coalition to NIH Office of Data Science Strategy (https://globalbiodata.org/, https://datascience.nih.gov/data-ecosystem/biomedical-data-repositories-and-knowledgebases). To help justify the funding of these data resources various impact metrics are collected by the resources and sometimes by funders (Imker 2018). Impact metrics can be used to aid assessment of the value of resources and also guide future directions of development of them. Staff at the UniProt database (UniProt Consortium 2023), a large database of protein sequence and annotation, have had many years of experience in calculating metrics, and we have developed a variety of novel metrics which are not widely available. In this work, we have developed a tool to share the methodology to calculate these metrics with other resources. The tool is not aimed to be a comprehensive resource for metrics, but rather provides a suite of information that is not readily available elsewhere. For example, citation information about the papers describing database resources are easily obtained from Google Scholar or a number of other similar resources. Website analytics is also an area that is well provisioned with metric gathering tools. Our tool named MBDBMetrics, for Molecular Biology DataBase Metrics, is a freely available tool that is hosted in Google Colab. This makes it easily accessible and it allows it to be readily modified by others. Alternatively, it can be downloaded as a jupyter notebook from the GitHub code repository, allowing it to be run on a user’s computer, independently of the google ecosystem.

## 2 Tool description

**Figure 1.**
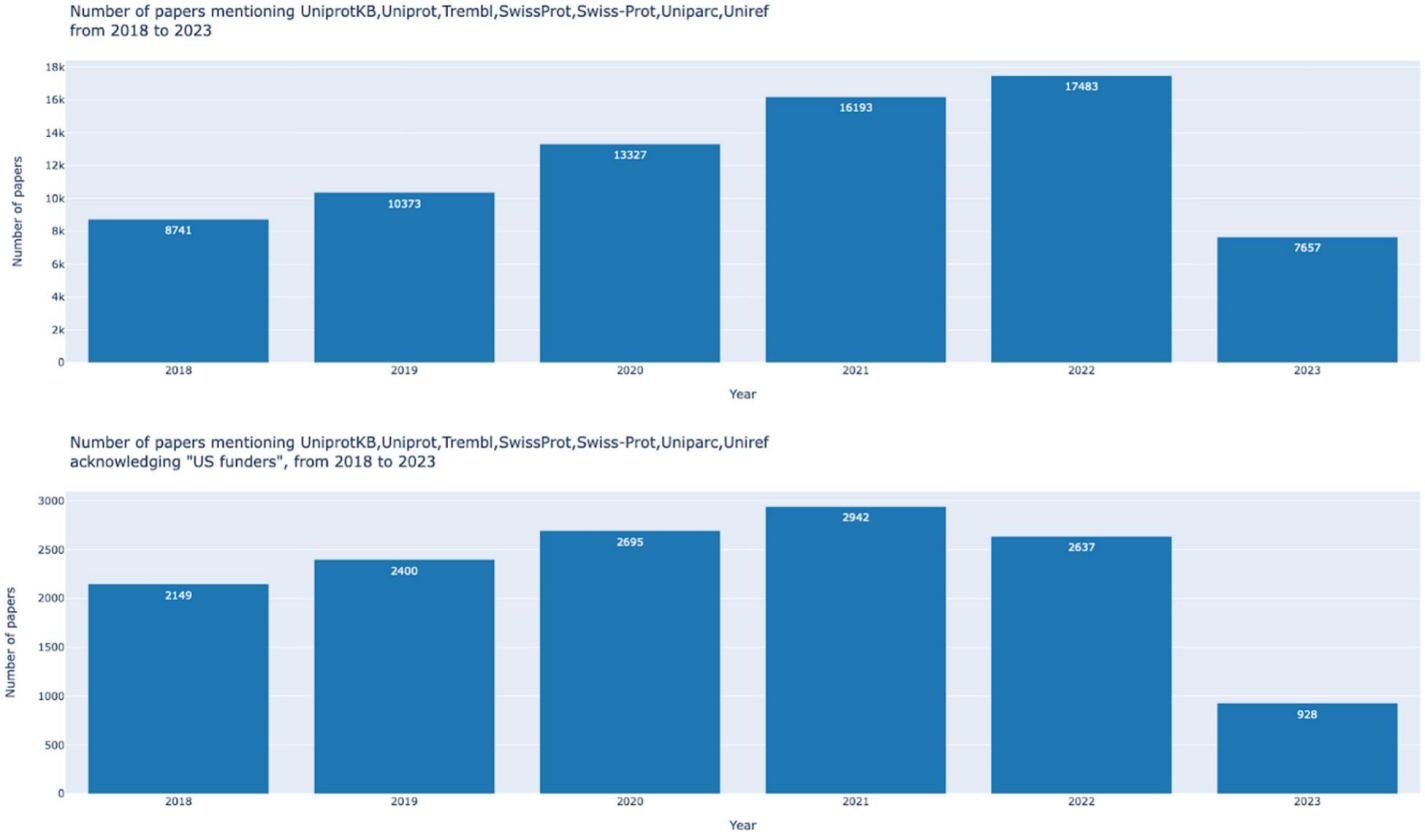
Example of the plot ‘Publication mentions by year’ for the date range 2018–23 and the resource UniProt (and its related keywords). Top panel: total number of papers for each year; bottom panel: restricted to those acknowledging ‘US funders’. More plot examples are available on the GitHub repository page.

MBDBMetrics was implemented as an interactive Jupyter notebook, coded in Python and depending only on the libraries *requests* (for web data retrieval), *plotly* (for creating the bar plots), and *pandas* (for exporting the datasets). The structure of the notebook consists of an initial Code cell (where the procedures are defined), a Parameters cell (where users can select the query strings, the date range, the grant funding agencies and the plots’ colour scheme) and a series of Plot cells, each of which is specific to generate a certain type of diagram. In this way, users have the possibility to only retrieve data and generate the plot(s) for the data dimension they are interested in. Once the parameters have been specified, the databases EuropePMC (Ferguson *et al.* 2021) (for mentions in publications) and SureCHEMBL (Papadatos *et al.* 2016) (for mentions in patents) are queried via their respective APIs. The retrieved data are then plotted and also made available for download in comma separated tabular format. This allows the possibility of plotting the data using any other external tool. A series of default parameters are accessible via drop down menus, but users can simply type in any value, without being limited to the preset choices. Since each Colab session runs independently and is saved in a user’s Google Drive, their own customizations will be preserved across runs. The Colab platform makes it easy for users to experiment with modifying and extending the code, so that it is easy not only to customize the search parameters, but also to add new capabilities and new plots. Similarly, users can clone the GitHub code repository and freely modify the original notebook in their own computer to extend its functionality or customize its output. The current version of MDBDMetrics generates the following plots:

- Total results (number of papers and number of patents mentioning the specified resource, in the specified time frame and/or acknowledging a certain funding agency). This graph gives an overview of the major metrics calculated by this tool.
- Publication mentions by year (number of papers mentioning the resource for each year in the specified range, with or without acknowledging a specified funding agency): see example in Fig. 1 below.
- Publication mentions by grant agency (a total of 28 funding agencies are currently defined but, again, it is easy to modify the code to change or extend this set). This plot enables an understanding of the relative use of a resource by fundees of different agencies.
- Publication mentions by paper section (18 paper sections are defined). This graph can be useful to highlight for example whether a resource is mostly mentioned in the Methods rather than in the Discussion or in the Abstract of publications.
- Patent mentions by year (number of patents mentioning the specified resource in the specified time frame). This graph can help to understand how a resource is used in records of new inventions and give a glimpse of industrial use.

There are some important caveats to note when using the tool. Firstly, to find all mentions of the data resource it is important to provide synonyms. Each search term must match exactly, thus ‘UniProt’ and ‘UniProtKB’ will match different sets of papers. If the name of the database matches a common English word such as STRING or PRINTS then you will get many false positive matches. A final caveat is that this tool is only searching the full text of the open access portion of EuropePMC, thus the counts of papers will be an underestimate of the total number of mentions in the whole research literature.

## 3 Comparison with existing tools

Currently, the two major metrics for demonstration of a molecular biology database’s impact is via citations to an article describing the resource or the number of website visits or users often from Google Analytics. Many database resources are described in the Nucleic Acids Research’s Database Issue and the most impactful publications gain thousands of citations making them among the most highly cited papers in all of science (Wren 2016). Citation impact can be easily found for any published paper by inspecting data at sites like Google Scholar, Thompson Reuter’s ISI Web of Knowledge or Elsevier’s SCOPUS. Although commonly used, the value of citation data has been questioned for many reasons (MacRoberts and MacRoberts 2018). Information about the number of website visits is available to the developers of the resource through tools such as Google Analytics or by analysing web log files directly. There are various challenges to interpreting this kind of data, such as the many papers which may use resources in their work but not cite them. With website analytics the challenges lie in separating out real users from web crawling robots and identifying users uniquely in the data. However, these issues are beyond the scope of this article.

We believe that our tool provides a unique and useful combination of metrics to be used alongside existing tools. One of the advantages of our tool is that, rather than relying on citations, we rely on mentions of a resource in the full text of the article. This means that we capture many uses or mentions of a resource that are missed by only counting citations. Having said that, not all articles are available as full text, so these two approaches should be regarded as complementary. In terms of patent searches, one can use the SureChEMBL database directly, but this does not return a result that is grouped by year, so it is not easy to see the growth of patent mentions over time as with our tool. Finally, we are not aware of any other tool that combines grant funding information with database mentions. This enables a better understanding of how much overlap there is for a particular resource with the community of researchers being funded (see case studies in Supplementary Materials).

Although the tool was developed in the context of database metrics, the tool can actually be used in a more general way. For example, one could look at the metrics for molecular biology methods such as CRISPR or RNAi, to judge their impact on the research field.

## 4 Conclusion

Molecular biology databases provide a foundation for much of modern biomedical research, saving researchers untold thousands of hours in their research. As collections of data gathered over decades and thousands of unique depositors, databases often do not just save time, they literally enable research that could not be done without a comprehensive database, no matter how many hours available. However, long-term stable funding for these resources is rarely available, meaning that recurrent grant funding applications are the norm (Markosian *et al.* 2018, Imker 2020). We hope that MBDBMetrics will be a user-friendly online tool for database developers to present the impact of their resources as well as a useful source of information for funders and other interested parties to evaluate database resources.

Although we think that MBDBMetrics is immediately useful, there are undoubtedly improvements that can be made over time. Currently the graphs presented in the Colab page are not of publication quality and hence we provide access to the raw data so the researchers can import and generate graphics to fit their own needs. Making higher quality graphs directly available via Google Colab could save researchers time. Currently the grouping of funders is US/UK centric. We are happy to take suggestions of further funder groups to be included within our tool to help justify database resources at the national or international level. Other detailed metrics on the topics of papers mentioning a resource could be included in the future inspired by the detailed analysis carried out by the RCSB PDB database (Markosian *et al.* 2018), although the subject categories used from the ISI database are not freely available data. We are open to further suggestions for improvements or collaborations on the tool to enhance its utility. We encourage database developers as well as funders to use MBDBMetrics alongside other tools to make the case for future stable funding of resources to ensure the long-term success of biomedical research.

## Supplementary Material

vbad180_Supplementary_Data
