# Supplementary material for: MBDBMetrics: an online metrics tool to measure the impact of biological data resources
Source: Bioinform Adv. 2023 Dec 18;3(1):vbad180. doi: 10.1093/bioadv/vbad180 (PMC10733715; doi:10.1093/bioadv/vbad180)
Supplement: vbad180_Supplementary_Data [file vbad180_supplementary_data.pdf]

Supplementary materials

User guide

To access the MBDBMetrics Tool's Colab follow this URL link (https://colab.research.google.com/drive/1aEmSQR9DGQIZmHAIuQV9mLv7Mw9Ppkin) in your favourite browser. The tool should work well on any modern browser.

The resulting Google Colab page is already pre-populated with result data for the UniProt database, meaning that users can immediately see the results of a specific run of the tool. The page contains introductory sections that briefly outline what metrics are available and how to run the tool.

To run the tool, first users need to execute (or "run") the Code "cell" (this is the usual term to indicate the units/blocks of an interactive coding environment) by pressing the "play" button located to the left of the cell. The button is shaped similarly to a "media play" icon (▶). Executing the cell is needed to initialise the code procedures needed for the tool's operation.

Below the Code cell, users will find the Parameters cell, which contains a form, shown in Figure S1. This cell also needs to be executed.

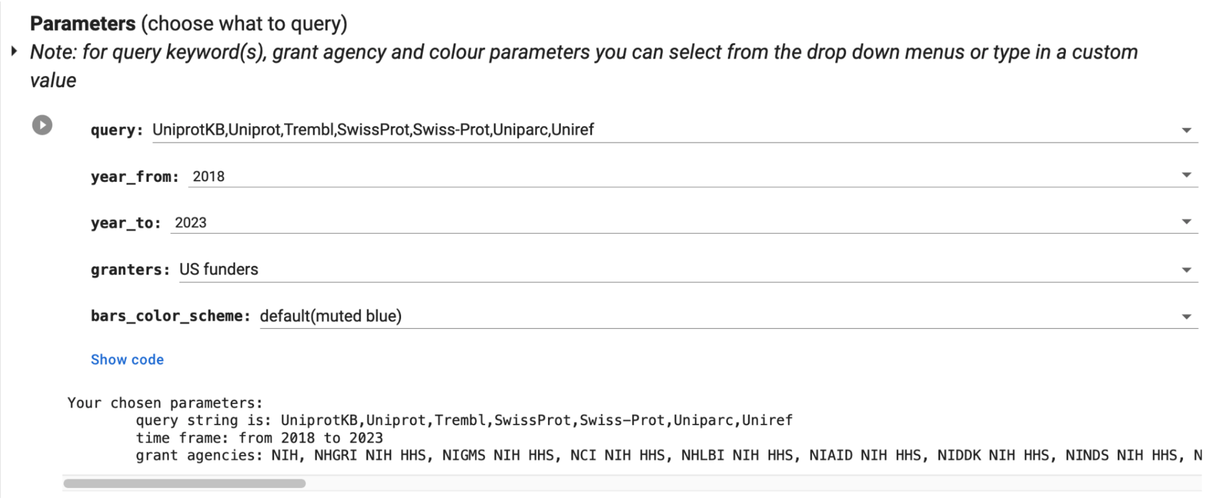

Figure S1. A screenshot of the parameters section of the MBDBMetrics tool.

Description of the input fields

The **query** field accepts a comma separated list of terms that will be used to search for matches in the literature and patent databases. In the pictured example, multiple search terms of often used synonyms for UniProt are listed. It is important to note that these terms are used for a simple text search and so if a database term is also the same as a common English word the tool will overestimate the number of mentions. For example, querying for the term "STRING" or "PRINTS" for the respective database would likely produce inaccurate results. It is also important to note that the search terms must match the whole work. The search term

UniProt will not match the word UniProtKB in the full text of papers. Thus, including synonyms will help to maximise the number of matches.

Users can select from the query field's dropdown menu a different set of search terms from a predefined list, or they can alternatively click on the list of terms and replace them with their own. A quick browse through the predefined choices should give an indication of the type of search keywords that can be used and how these could be adapted to the specific resource the users are interested in querying.

The second and third field in the form allow to specify a time frame for the search of publication and patent mentions.

The **granters** field is used to select a specific funding agency or a collection of them (for example "US funders" or "UK funders" - whose definitions are contained in the code and can be freely modified). Analogously to the query field, users are not limited to the predefined choices found in the dropdown, but can freely type a custom value. Obviously the name inserted in this field should correspond to an existing funding agency in order to obtain sensible results.

Finally, it is possible to select a different colour scheme for the plots.

Provided the Parameters cell has been executed at least once, all changes to the input fields will be automatically applied, as indicated by the text shown in the "output block" of the Parameters cell (the one starting with "*Your chosen parameters:*"), which should update at each change of the input fields.

Once the Parameters have been selected, the users can start to generate plots corresponding to the specified input. It is not necessary to proceed sequentially and generate all the plots: the users can scroll down to the diagram which visualises the kind of metrics they are interested in, and execute its corresponding code cell.

For example, a user can scroll down to the cell named "Generate plot by paper section" and execute it. The tool will at this point start querying the publication database for mentions of the specified search terms, in the specified time frame and - once the information has been fully retrieved - plot it according to the paper sections.

Continuing with this example workflow, the user could go back to the Parameters section and change the time frame, or maybe the search terms, and then scroll down once more to re-generate that particular plot according to the new parameters.

Description of the plots interface

The generated plots have an interactive interface (provided by the *plotly* library), allowing users to zoom in, select specific sections and even draw over the plot, all through a series of buttons from a toolbar located at the top right corner of each plot. One of those buttons also allows the plot to be downloaded as a png file.

Finally, under each plot, a link is provided to download the raw data, as a comma separated value file. This file can then be read in and used to recreate the diagram using any external plotting tool.

Case studies

Pfam Case Study

As an example use case we investigate the highly used and cited Pfam database. We set the parameters: Query to 'Pfam' and years from 2013 to 2023 and Granters to 'UK funders'. From the graphs in Figure S2 we can see easily that there has been a strong growth of papers mentioning Pfam over the last 10 years, (Figure S2A), that the number of patents mentioning Pfam is about 3-400 per year (Figure S2B). We can also see in Figure S2C that the major UK funders for the users of Pfam are the BBSRC, Wellcome Trust and the Medical Research council. Finally, we can see that Pfam is mentioned most often in the reference, results and methods section which fits with our expectation of Pfam being a highly cited tool for finding protein families and domains.

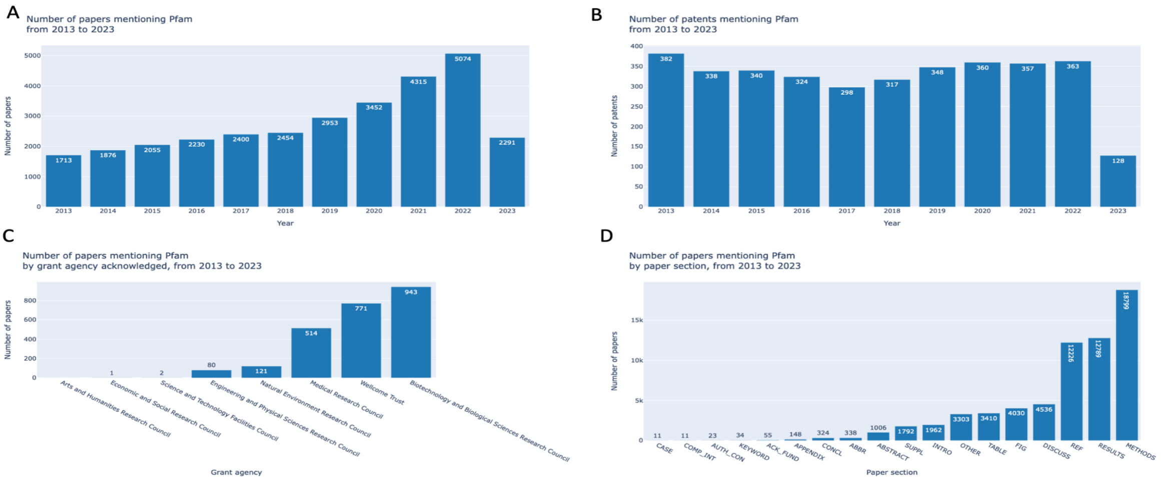

Figure S2. Selected graphs from MBDBMetrics tool for the Pfam database.

Cas9 Case Study

To illustrate that the MBDBMetrics tool is not restricted to only molecular biology resources we show that we can replace the query with any word or phrase. In this case we selected the popular genetic modification tool CRISPR-Cas9 using the query term Cas9. In figure S3 we show the steep growth in mentions of Case 9 over the last decade and the graph hints that we may be close to saturation with the 2021 and 2022 number of mentions being very close at around 18,000.

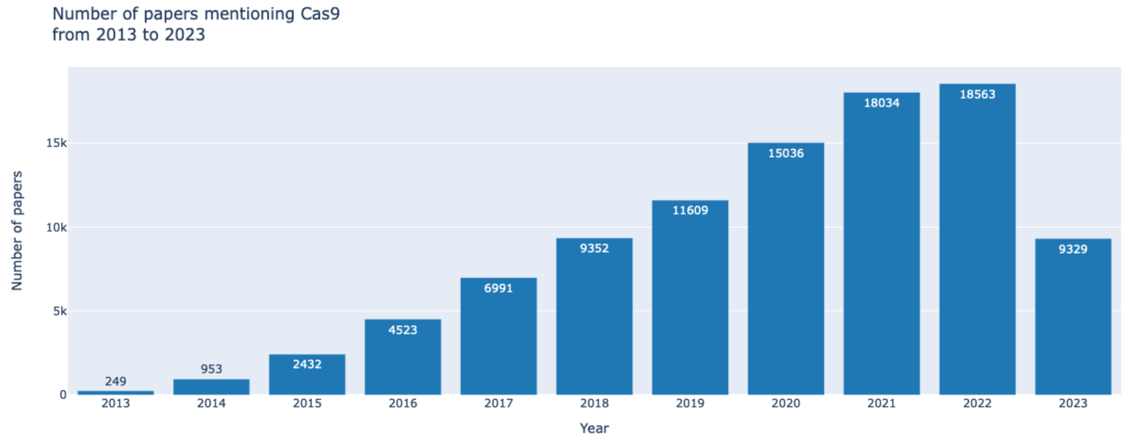

Figure S3. A graph showing the growth of mentions of Cas9 in the full text literature of Europe PMC.

MGnify communities use case

In this example we can look at the mentions of the MGnify metagenomics database by funding acknowledgements to UK funders. The results found in Figure S4 show that the major UK funder acknowledged with respect to MGnify is the BBSRC agency.

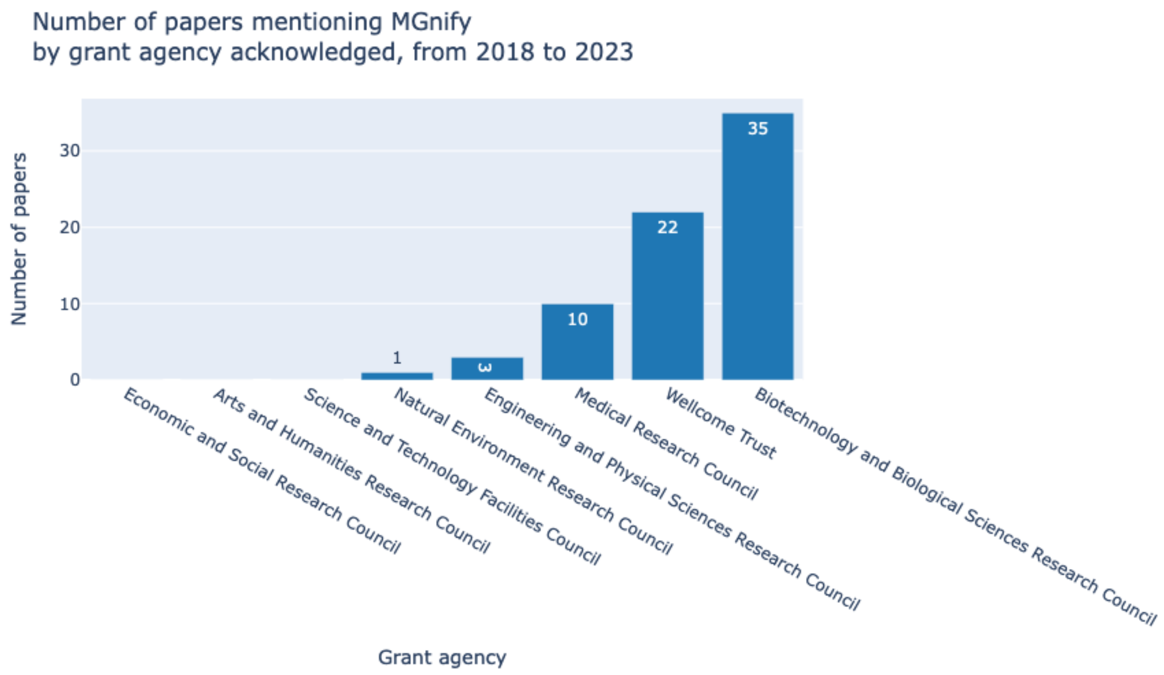

Figure S4. A graph showing the number of mentions of MGnify database in papers acknowledging UK funders.
